# Coordination Pattern Adaptability: Energy Cost of Degenerate Behaviors

**DOI:** 10.1371/journal.pone.0107839

**Published:** 2014-09-25

**Authors:** Ludovic Seifert, John Komar, Florent Crettenand, Grégoire Millet

**Affiliations:** 1 Centre d'Etude des Transformations des Activités Physiques et Sportives (CETAPS) - EA 3832, University of Rouen, Faculty of Sports Sciences, Mont Saint Aignan, France; 2 ISSUL Institute of Sport Sciences - Department of Physiology, Faculty of Biology and Medicine, University of Lausanne, Lausanne, Switzerland; Ludwig-Maximilian University, Germany

## Abstract

This study investigated behavioral adaptability, which could be defined as a blend between stability and flexibility of the limbs movement and their inter-limb coordination, when individuals received informational constraints. Seven expert breaststroke swimmers performed three 200-m in breaststroke at constant submaximal intensity. Each trial was performed randomly in a different coordination pattern: ‘freely-chosen’, ‘maximal glide’ and ‘minimal glide’. Two underwater and four aerial cameras enabled 3D movement analysis in order to assess elbow and knee angles, elbow-knee pair coordination, intra-cyclic velocity variations of the center of mass, stroke rate and stroke length and inter-limb coordination. The energy cost of locomotion was calculated from gas exchanges and blood lactate concentration. The results showed significantly higher glide, intra-cyclic velocity variations and energy cost under ‘maximal glide’ compared to ‘freely-chosen’ instructional conditions, as well as higher reorganization of limb movement and inter-limb coordination (p<0.05). In the ‘minimal glide’ condition, the swimmers did not show significantly shorter glide and lower energy cost, but they exhibited significantly lower deceleration of the center of mass, as well as modified limb movement and inter-limb coordination (p<0.05). These results highlight that a variety of structural adaptations can functionally satisfy the task-goal.

## Introduction

Dexterity was defined by Bernstein [1] as the expert's ability to reach the goal of a task correctly (movement outcome), quickly, rationally (movement organization), efficiently and with resourcefulness. However, for Bernstein, the heart of dexterity refers to resourcefulness that relates to two important properties reflecting passive and active adaptation; i.e. ‘stability’ and ‘initiative’, respectively [1]. Stability enables the performance of movements to solve problems despite external, perturbing influences. According to Bernstein [1], it also helps to « *find those adaptive switchings that save motor act from destabilization and deautomatization, and the motor task from disruption when external changes and unexpected events occur* » (p. 221). Beyond passive adaptation to external disturbance, Bernstein [1] defined active changes in the movement processing (i.e., initiative), as the ability to search a route for an optimal result. Thus, dexterity can be considered as the ability to solve a problem quickly and in all situations. In other words, dexterity does not refer to movements themselves, but the ability to adapt to external constraints. In the past, Johnson [2] has already defined expertise as the combination of speed, accuracy, form (economy) and adaptability. An adaptive skill means that performance is proficient under varying and even unpredictable constraints [2]. By highlighting the importance of adaptability to define expertise, Johnson [2] already questioned the status, role and importance of the movement variability. Research in ecological dynamics has shown that movement system variability should not always be considered as noise detrimental to performance, error, or deviation from the expert model, to be corrected by the beginner [3]. In considering movement variability as functional involves exploring the meaning of adaptive behavior. Adaptability relates to an appropriate ratio between stability (i.e., persistent behavior) and flexibility (i.e., variable behavior) [4]–[7] and is essential to produce skilled performance in sport. On one hand, behavior is characterized by stable and reproducible movement patterns and coordinative structures. These patterns are stable in the sense that the functional form of movement is consistent over time, resists to perturbation and is reproducible; e.g. a similar pattern may recur on different task and environmental constraints. On the other hand, behavior is not stereotyped and rigid but flexible and adaptive. Even if movement patterns could show regularities and similarities within their structural components, an individual is not fixed into a rigidly stable solution but can adapt his movement pattern in a functional way. Ranganathan and Newell [8] defined flexibility as the “*ability to use different solutions to achieve the task-goal under task conditions when a certain subset of solutions is no longer viable* » (p. 756). We defend a model of expertise that articulates stability and flexibility: experts and non-experts have their own stable states and sometimes share the same coordination patterns; however, the characteristics of experts are their capacity for adaptability, i.e. to be stable when needed but variable depending of varying conditions [5]. In fact, although the human system naturally tends to become stable and more economical [9], stability and flexibility are not opposite. Notably, flexibility is not a loss of stability but conversely is a sign of adaptability [6], [7]. In relation to the previously cited definition of dexterity by Bernstein, the economy and efficiency of an adaptable pattern could be a key feature of expertise. Therefore, the last stage of motor learning was called ‘skill’ by Newell [10], which corresponds to an optimization of the coordination pattern. Optimization refers to the energy or mechanical economy and/or efficiency that individuals are able to produce by exploiting the passive, inertial and mechanical properties of body segments in order to perform economical and fluid movement [9], [11].

In bimanual coordination task [12], a traditional way to assess coordination stability and flexibility is to scan the intrinsic dynamics of an individual, i.e., by increasing linearly the control parameters (e.g., frequency of the finger oscillations) and by instructing an individual to adopt both in-phase and anti-phase coordination patterns of finger flexions-extensions. In human locomotion, a similar scanning task has been conducted by increasing linearly treadmill speed to assess limb coordination through walking-running gait transition [13]. A similar protocol was adapted in swimming to scan the intrinsic coordination dynamics [14]. In parallel to running, swimming is a cyclic task, where swimmers have performed numerous cycles during training and competitions, expertise cannot be reduced to the ability of repeating an idealized movement pattern in an identical way from cycle to cycle, lap to lap and race to race, but rather the achievement of adaptive coordination solutions in dynamic environments [5]. Indeed, because the arm and leg recoveries are underwater, breaststroke exhibits the highest aquatic resistances among the four competitive swimming strokes, requiring a continuous management of inter-limb coordination to maintain both the most hydrodynamic position [15], [16] and, in the same time, to generate the highest propulsion possible. In a recent study, it was shown in expert swimmers that the nature of the arm-leg coordination as well as the acceleration of the center of mass is not necessarily affected by the swim speed during leg and arm propulsion [17]. In fact, when swim speed changed, swimmers mainly adapted the glide duration [16], [17], reflecting in accordance with Chollet et al. [15] who differentiated between ‘glide’, ‘continuous’ and ‘superposition’ breaststroke techniques. However, the relationships between motor adaptations and the efficiency and energy cost of locomotion remain rarely analyzed, mainly because increasing glide duration has been traditionally associated with higher intra-cyclic velocity variations (*IVV*) of the center of mass [18], [19] and higher energy expenditure [20]–[22].

The aim of this study was therefore to investigate motor adaptability in relation to changes in energy cost in a stable swim speed condition. We sought to highlight (i) how swimmers adapt their limbs' movement and/or inter-limb coordination (i.e., which phases of the movement within underwater recovery, glide and propulsion are kept stable or are flexible) when they are instructed to modify their behavior, and (ii) if/how this motor adaptability could impact *IVV* and energy cost. We hypothesized that (i) expert swimmers developed skills to adapt their behavior, and (ii) the behavior leading to minimal *IVV* would be the most economical one.

## Methods

### 1. Participants

Seven national level swimmers, specialists in breaststroke participated voluntarily in this study. Expertise level was expressed in percentage of the current world record (W.R.) of their best performance for 200-m breaststroke. The mean ± standard deviation of age, weight, height, arm length, performance in 200-m and expertise were: 17.5±2.2 yrs, 61.7±8.1 kg, 1.75±0.07 m, 0.58±0.02 m, 161.8±8.7 s, 88.7±3.0%W.R., respectively.

### 2. Protocol design

Testing as carried out in a 50-m indoor pool, after a moderate intensity individual warm-up and broadly involved a steady intermittent procedure. Specifically, the swimmers performed three consecutive 200-m trials at 70% of their breaststroke 200-m personal best time as recorded within the month preceding the testing period. This percentage was chosen with intention to achieve a target speed of 0.91 m.s^−1^. This intensity and work duration were selected to enable the swimmers to finish each trial in greater than 3 min 30 s, requiring they reach a VO_2_ steady state [23]. In the first trial, swimmers were instructed to swim using a ‘freely-chosen’ coordination pattern. In trials 2 and 3 either (randomized) swimmers were instructed to either ‘maximal glide’ or ‘minimal glide’ between each consecutive propulsive action. Five minutes rests were allowed between trials. Swimming speed was monitored by an experimenter walking along the edge of the pool while holding a stick immersed in front of the swimmers head. Visual markers were placed every 2.5 m along the edge of the pool. Using an Aquapacer ‘Solo’ (Challenge and Response, Inverurie, UK), the experimenter walked along the edge of the pool, so he could match auditory signals with visual markers. The experimenter simulated on 50 m the pace of each trial to guarantee accuracy in the swimming speed pacing. The swimmers were then asked to follow this stick handled by the experimenter. A distance of one meter was accepted between the stick and the swimmer. When this distance was overcome, the swimmer was verbally encouraged to bridge the gap. When the swimmer could not bridge this gap, the trial was stopped. It can be noted that, the swimmers were rarely further than one meter from the stick and they were never unable to bridge this gap. White body markers were placed bilaterally on each swimmer on the anatomical landmarks of the wrist (radiocarpial joint), elbow (ulnohumeral joint), shoulder (humeral head), hip (greater trochanter of the femur), knee (tibiofemoral joint) and ankle (talocrural joints).

### 3. Ethics statement

This study was performed in accordance with the Declaration of Helsinki and was approved by the Institutional Review Board and Ethics Committee of the Faculty of Biology and Medicine, University of Lausanne (protocol #87/10). Procedures were explained by a written document to the participants and to their parents, who then gave their written informed consent to participate. Parents or caretakers gave written consent for minors enrolled in this study.

### 4. Video recording

According to previous studies [24], [25] a calibration frame of 6-m in horizontal-axis (X), 3-m in vertical axis (Y) and 2-m in lateral axis (Z) was positioned on the floor of the pool, in the first lane and orthogonal to the wall. Two aerial and four underwater (1.0-m) fixed side-view cameras (50 Hz) were positioned on one side of the calibration frame (angle between adjacent cameras varied between 100 and 110°). Video cameras recorded two stroke cycles per swim taken in the central part of the pool, this during the last 100-m of the trial (total number of cycles, *n* = 4). Fields of view of the cameras were overlapped to ensure that all the body markers were within the view of at least two cameras at any time. One stroke cycle corresponded to the period from one maximal knee flexion to the next maximal knee flexion. The six views were synchronized and genlocked a posteriori with Adobe Premiere 6.0.

### 5. Angle measurements and arms-legs coordination

Digitization of body markers on video data allowed 3-D reconstruction of body markers using APAS software (Ariel Dynamics) and allowed the calculation of relative elbow and knee angles. As previously done [24], [25], error of digitizing was assessed by calculating the root mean square (RMS in mm) and the coefficient of variation (CV in %) of 10 digitizations of the same individual. The calculated error of digitizing was: in X RMS  = 2.4 mm, CV  = 0.48%; in Y RMS  = 2.73 mm, CV  = 0.81%; in Z RMS  = 2.98 mm, CV  = 1.35%). Trunk inclination (in the X, Y plan) was calculated as the angle between the water surface (Y = 0) and the trunk of the swimmer characterized by the hip-shoulder segment. Angular displacements of knee and elbow were calculated as the arctangent of the dot product of the limb unit vectors of two adjacent limbs. Standard corrections for quadrant were applied in order to ensure that angles were correct. Continuous angular velocities were then computed as the first derivative of the angular position using the central difference formula. Arm-leg coordination patterns were assessed using continuous relative phase (*φ*_rel_, in degrees) between two oscillators (i.e. elbow and knee angles). In accordance with Hamill et al.[26], the data on angular displacements (*θ*_norm_) and angular velocities (*ω*_norm_) were normalized in the interval [−1, +1]. Knee and elbow angles were filtered using a low-pass Fourier filter (cut-off frequency 6 Hz). Then phase angles (*φ*_elbow_ and *φ*_knee_, in degrees) were calculated and corrected according to their quadrant [26]: 
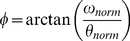
(1)


Finally, the continuous relative phase for a complete cycle was calculated as the difference between both phase angles [26]:
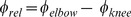
(2)


Theoretically, two extreme patterns of coordination are possible: in-phase (*φ*_rel_  = 0°) and anti-phase (*φ*_rel_  = 180°); however, following previous studies [13], [27] on inter-limb coordination, a lag of ±30° was accepted to define the adopted coordination pattern. Therefore, an in-phase pattern was assumed to occur when −30°<*φ*_rel_>30°, while an anti-phase pattern was defined by −180°<*φ*_rel_>−150° and 150°<*φ*_rel_>180°. According to Seifert et al. [16], [27], several features of *φ*_rel_ through a cycle were explored: (i) The mean and standard deviation of *φ*_rel_ through a cycle indicates the main pattern of coordination between limbs and how this coupling varies through the cycle. (ii) The first *φ*_rel_ value of the cycle defines the capability of the swimmers to synchronize knees flexion with arms extension. A value close to −180° (i.e. anti-phase relationship) indicates that the elbows are at their maximal extension when the legs are at their maximal flexion prior starting their extension. A value closer to 0° indicates that the elbows are flexed when the knees are at their maximal flexion. (iii) The time spent in in-phase pattern of coordination indicates an identical motion of both arms and legs (i.e. flexion or extension of both pairs of limbs). For instance, the time spent in simultaneous extension of arms and legs indicates the body glide duration. (iv) The maximal peak of *φ*_rel_ identifies the period when the legs start their recovery whereas the arms are extended.

### 6. Intra-cyclic velocity variations of the center of mass and stroking parameters

According to previous studies [18], [21], [22], four key points of the cycle were selected to assess the *IVV* of the center of mass: (i) Maximum velocity of the center of mass achieved at the end of leg propulsion (Max_Leg_), (ii) Maximum velocity of the center of mass at the end of the arm propulsion (Max_Arm_), (iii) Minimum velocity of the center of mass during the transition between arm and leg propulsion (Min_Transitional_), which corresponds to velocity of the center of mass while the body glides in fully the extended position, (iv) First minimum peak of the center of mass velocity (Min_Leg_) following arm and leg recovery and corresponding to the beginning of leg propulsion. The instant position of the center of mass was based on the anatomical model adapted by de Leva [28]. Six anatomical points were digitized and the head was therefore considered as fixed relatively to the trunk, the feet as fixed relatively to the shanks, and the hands as fixed relatively to the forearms [29]. Instantaneous velocity of the center of mass was calculated based on the displacement of the center of mass in the swimming direction. The *IVV* of the center of mass was used as an indicator of swim efficiency [18], [20]–[22] and was calculated on the basis of acceleration-deceleration through the cycle:
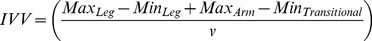
(3)where *v* is the mean swimming velocity of the center of mass during a cycle (in m.s^−1^).

The traditional stroking parameters were calculated for each cycle: *v*, the stroke rate (SR, in Hz, defined as 1/cycle duration) and the distance per stroke (SL, in m).

### 7. Energy cost of locomotion

During exercise, minute ventilation (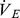), oxygen consumption (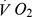) and carbon dioxide production were recorded breath-by-breath with the K4b2 metabolic card and AquaTrainersnorkel (COsmed, Rome, Italy) [30], [31] which was calibrated according to the manufacturer's instruction before each test. Etopic artefacts were manually eliminated; then data were 5-sec averaged. A fingertip capillary blood sample was obtained at rest and no more than 30-sec after the end of each trial as well as three min after the last trial and analyzed for blood lactate concentration (lactate Pro LT, Arkay Inc., Kyoto, Japan). However such procedure does not guarantee that we measured the peak lactate. Therefore, the glycolytic contribution might be slightly underestimated. The energy cost (C) of locomotion (mLO_2_.kg^−1^.m^−1^) was defined as [32]:
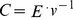
(4)


Where 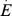 is the total metabolic energy expenditure (aerobic and anaerobic pathways) expressed in mLO_2_.min^−1^.kg^−1^ and *v* (in m.min^−1^) is the swimming speed. The aerobic part of swimming *C* (*C*_aero_) was equal to the ratio between net 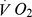 (i.e. the difference between the
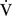
O_2_ measured during the last minute of each swimming trial and its value at rest) and the swimming speed [32], [33]. *C*_aero_ was calculated over the last 100-m of the 200-m trial where there was a steady state of 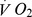 [32]. Anaerobic glycolytic net *C* (*C*_anaero_) was estimated using blood lactate. Net blood lactate measures (mmol) were converted to oxygen equivalent values as 3.0 mLO_2_.kg^−1^ of bodyweight per mmol of blood lactate [34]. Thus, *C*, calculated as the addition of *C*_aero_ and *C*_anaero_, represented the energy expended to cover one unit of distance while swimming at a given speed and with a given stroke. Following Rodriguez [35], anaerobic alactic energy sources were neglected. Finally, *C* was expressed in J.kg^−1^.m^−1^ assuming that 1 mLO_2_ yields 20.9 J [32].

### 8. Statistical analysis

Mean and standard deviation were computed for the trunk inclination, the knee-elbow angles and φ_rel_, the maximal and minimal values of velocity of the center of mass, the *IVV* and the energy cost. Cycles were time-normalized (100%) allowing comparison and averaging between participants (4 cycles ×7 participants ×3 conditions, *n* = 84). The distribution was tested for normality (Ryan Joiner test) and homogeneity of variance (Bartlett test). A one-way repeated measure ANOVA was conducted using SPSS Statistics 20.0 to compared the three conditions. Compound symmetry, or sphericity, was verified by the Mauchly test [36]. When the assumption of sphericity was not met, the p-value was adjusted according to the Greenhouse-Geisser procedure. Then, False Discovery Rate (FDR) correction across all the ANOVA condition main effects was done according to Benjamini and Hochberg [37]. Then, post-hoc pairwise condition comparison Bonferroni tests were applied where the main effect was significant by the FDR. Finally, familywise error rate was controlled by applying a Bonferroni correction of the p-value [38]. Partial eta squared (η_P_^2^) was calculated as an indicator of effect size, considering that η_P_^2^ = 0.01 represents a small effect, η_P_^2^ = 0.06 represents a medium effect and η_P_^2^ = 0.15 represents a large effect [39]. For all tests, the level of significance was fixed at p<0.05.

## Results

### 1. Stroking parameters

There were no significant differences between the target and the recorded swimming speeds (average speed equals 0.90±0.07 m.s^−1^). Moreover, there were no significant differences in swimming speeds between the three coordination conditions (Table 1), supporting the accuracy of the pacing and task achievement in terms of speed. However, there were differences in stroke rate and stroke length between the three coordination conditions (Table 1).

**Table 1:** Stroking parameters, angles, arms-legs coordination, intra-cyclic velocity variations, maximal and minimal values of velocity of center of mass and energy cost of locomotion in the three coordination conditions.

Parameters	Maximal glide		Freely-chosen		Minimal glide		ANOVA	ANOVA		Post-hoc tests
	*M*	*SD*	*M*	*SD*	*M*	*SD*	*F* value	*p* value	η_P_^2^	*p* value
Swimming speed (m.s^−1^)	0.90	0.08	0.90	0.07	0.91	0.06	*F*_2,26_ = 1.76	*p = *0.66	0.08	
Stroke rate (Hz)	0.40	0.07	0.49*	0.06	0.58*$	0.09	*F*_2,26_ = 36.82	*p*<0.0001	0.74	*$ *p<*0.0001
Stroke length (m)	2.24	0.23	1.84*	0.19	1.57*$	0.26	*F*_1.51,40.69_ = 57.05	*p = *0.006	0.71	*$ *p<*0.0001
Maximal trunk inclination (°)	38.4	6.4	42.2	9.0	41.3	8.4	*F*_2,26_ = 1.67	*p = *0.69	0.08	
Minimal trunk inclination (°)	2.9	5.9	8.2*	3.9	11.9*$	7.5	*F*_2,26_ = 47.83	*p*<0.0001	0.74	*$ *p<*0.0001
Mean trunk inclination (°)	14.8	4.5	21.6*	3.6	23.2$	5.3	*F*_2,26_ = 40.93	*p*<0.0001	0.79	*$ *p<*0.0001
Elbow angle at start of leg propulsion (°)	142.8	21.0	135.7	23.6	126.3*$	23.3	*F*_2,26_ = 14.69	*p*<0.0001	0.53	*$ *p<*0.0001
End of the leg extension (%)	15.2	2.7	19.2*	2.7	23.5*	5.2	*F*_1.55,41.85_ = 46.01	*p = *0.012	0.65	* *p<*0.0001
Start of leg flexion (%)	67.7	6.2	61.6*	6.7	57.3$	5.8	*F*_1.48,39.97_ = 12.77	*p = *0.004	0.62	*p = *0.001
End of arm extension (%)	8.0	4.4	15.3*	9.9	20.8*$	9.7	*F*_2,26_ = 24.92	*p*<0.0001	0.65	*$ *p<*0.0001
Start of arm flexion (%)	59.0	6.7	50.0*	4.6	43.8*$	8.5	*F*_2,26_ = 29.97	*p*<0.0001	0.69	*$ *p<*0.0001
Time of *φ_rel_* spent in in-phase coupling (%)	48.1	12.8	33.0*	9.4	36.0$	11.2	*F*_1.41,38.1_ = 20.08	*p = *0.001	0.53	*$ *p<*0.0001
Mean *φ*_rel_ through cycle	−29.6	−13.6	−38.3*	11.6	−45.2$	13.5	*F*_1.52,41.07_ = 17.17	*p = *0.007	0.66	*$ *p = *0.001
*SD* of *φ_rel_* through cycle	60.0	7.7	65.7*	7.3	56.1*	8.3	*F*_2,26_ = 37.08	*p = *0.001	0.74	* *p = *0.001
*φ_rel_* at the start of leg propulsion (°)	−129.1	40.6	−125.1*	46.2	−113.4$	42.4	*F*_2,26_ = 6.23	*p*<0.0001	0.32	* p = 0.013
										$ *p* = 0.004
Maximal *φ_rel_* (°)	34.3	12.7	43.0*	10.4	32.9*	15.1	*F*_2,26_ = 12.85	*p*<0.0001	0.49	* *p = *0.001
*IVV*	0.93	0.23	0.84	0.24	0.73$	0.21	*F*_2,26_ = 5.29	*p*<0.0001	0.29	$ *p* = 0.003
Max_Leg_ (m.s^−1^)	1.07	0.12	1.03*	0.09	1.04	0.09	*F*_2,26_ = 4.62	*p*<0.0001	0.26	* *p* = 0.005
Max_Arm_ (m.s^−1^)	1.10	0.10	1.11	0.10	1.12	0.10	*F*_2,26_ = 0.21	*p* = 0.74	0.02	
Min_Transitional_ (m.s^−1^)	0.75	0.09	0.84*	0.12	0.93*$	0.12	*F*_1.64,44.31_ = 44.24	*p*<0.0044	0.57	*$ *p<*0.0001
Min_Leg_ (m.s^−1^)	0.59	0.11	0.55	0.10	0.55	0.09	*F*_2,26_ = 2.44	*p = *0.29	0.11	
Energy cost (J.kg^−1^.m^−1^)	19.0	2.3	17.1*	2.7	17.8$	2.9	*F*_2,12_ = 8.66	*p*<0.0001	0.59	*$ *p<*0.0001

*M*: mean; *SD*: standard deviation; %: percentage of cycle duration; η_P_^2^: partial eta squared; *: significant difference from coordination condition in previous column with p<0.05; $: significant difference between ‘maximal’ and ‘minimal glide’ conditions with p<0.05.

### 2. Angle, arms-legs coordination and intra-cyclic velocity variations

The main result shows that leg propulsion and body glide phases were adapted when the swimmers were instructed to swim with ‘maximal glide’ or ‘minimal glide’.

**Figure 1:**
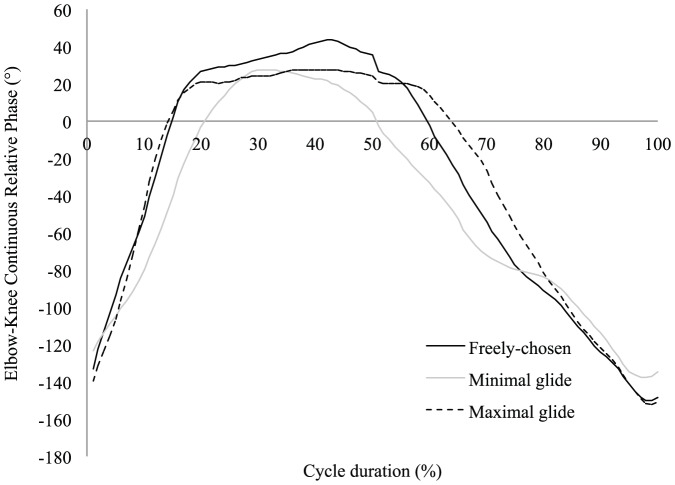
Elbows-knees continuous relative phase (mean of six cycles from all participants) for the three coordination conditions. One stroke cycle corresponded to the period from one maximal knee flexion (0%) to the next maximal knee flexion (100%).

**Figure 2:**
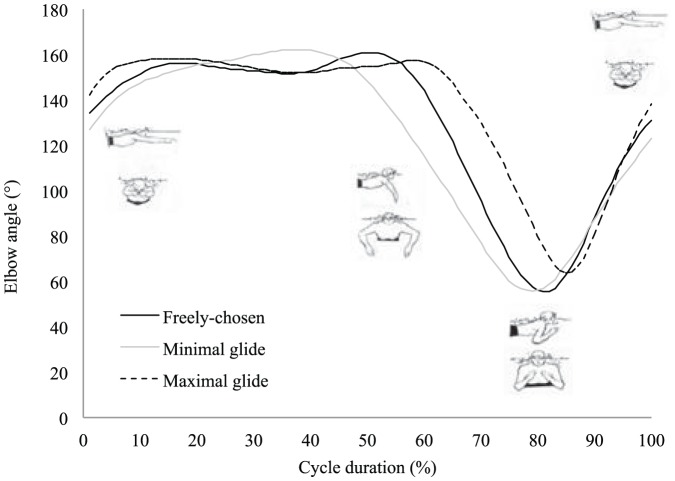
Elbows angle (mean of six cycles from all participants) for the three coordination conditions. One stroke cycle corresponded to the period from one maximal knee flexion (0%) to the next maximal knee flexion (100%).

**Figure 3:**
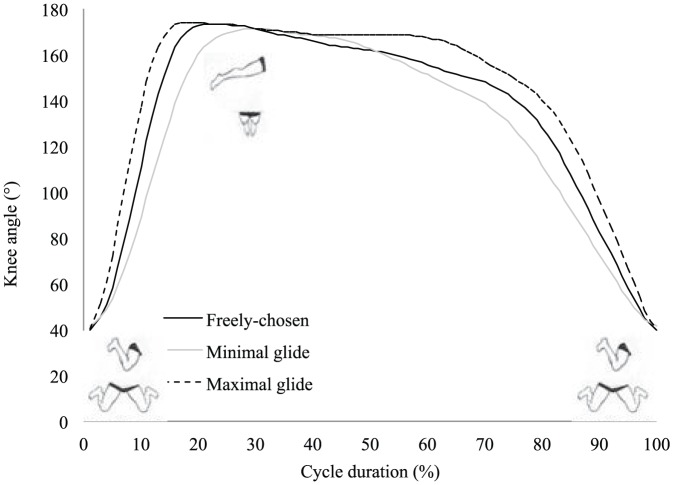
Knees angle (mean of six cycles from all participants) for the three coordination conditions. One stroke cycle corresponded to the period from one maximal knee flexion (0%) to the next maximal knee flexion (100%).

**Figure 4:**
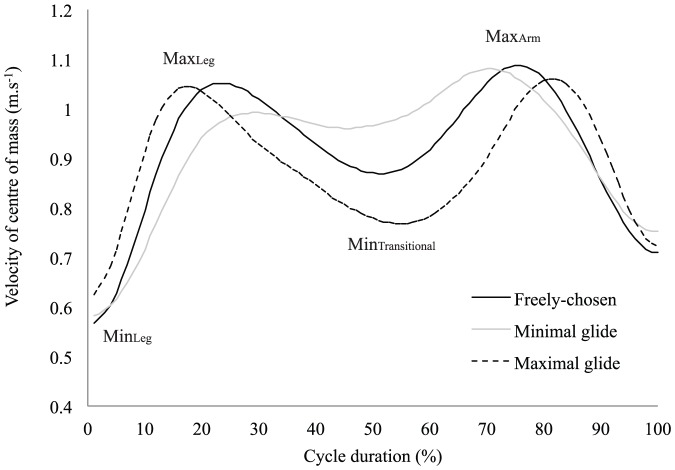
Instantaneous velocity of the center of mass (mean of six cycles) for the three coordination conditions.

The ‘maximal glide’ condition was characterized by flattest trunk position, with the lowest minimal angle of inclination regarding the horizontal axis (Table 1). This flattest trunk inclination suggests higher streamline body position that is supported by higher elbow extension and φ_rel_ value (indicating greater arm extensions whilst the legs are in maximal flexion) at the start of the legs propulsion and confirmed by an earlier end of full extension of the elbows (i.e., end of arm recovery) than in the other conditions (Figures 1 and 2, Table 1). In the ‘maximal glide’ condition, shorter relative duration of the legs propulsion was observed, so the end of the legs extension occurred earlier in the cycle than in the other conditions (Figure 3, Table 1). However, this was not detrimental to the legs propulsion outcome because the instantaneous speed of the center of mass (Max_Leg_ in Table 1) and the *IVV* were higher than in the other conditions (Figure 4). Moreover, in the ‘maximal glide’ condition, a longer relative duration of glide (i.e., time spent in in-phase pattern coupling) occurred, with higher inter-limb coupling in in-phase coordination pattern (see mean and maximal φ_rel_ value in Table 1 and Figure 1). This long glide duration was associated with a later start in the cycle of the elbows flexion (i.e., arm catch and propulsion) and a delay of the start of the legs flexion (i.e., leg recovery) (Figures 2 and 3, Table 1). However, this longer relative duration of glide was also associated to lower instantaneous speed of the center of mass (Min_Transitional_ in Table 1) than in the two other conditions (Figure 4).

In the ‘minimal glide’ condition, higher mean trunk inclination with higher minimal angle value of inclination were observed (Table 1). This higher trunk inclination may result from the constraint (e.g. lower glide duration requested by the operator) and suggests lower streamline body position that is supported by lower values of elbows extension and φ_rel_ at the start of the legs propulsion and late full extension of the elbows (Figures 1 and 2, Table 1). In the ‘minimal glide’ condition, the swimmers increase the relative duration of leg extension (i.e., longer leg propulsion) (Figure 3) without providing higher instantaneous velocity and acceleration of the center of mass (see Max_Leg_ and *IVV* in Table 1 and Figure 4). Interestingly, in ‘minimal glide’, the swimmers started earlier in the cycle their elbows flexion (i.e., arms catch and propulsion) (Figure 2) and exhibit higher transitional velocity of center of mass than in the two other conditions (see Min_Transitional_ Table 1 and Figure 4). Finally, in the ‘minimal glide’ condition, the swimmers started earlier their legs flexion (i.e., legs recovery) than in the other conditions (Figure 3, Table 1).

### 3. Energy cost of locomotion

The energy cost was significantly higher in ‘maximal glide’ than in the two other conditions (Table 1).

## Discussion

The aim of this study was to assess the inter-limb coordination adaptability by exploring how expert breaststroke swimmers are adaptable (i.e., what is kept stable or flexible when swimmers are instructed to vary the glide duration). The task-goal was only partially reached by the swimmers because when instructed to use a ‘minimal glide’, most of them were not able to glide less than in the ‘freely-chosen’ condition. However, our hypotheses were accepted for two reasons: (i) In addition to the modified glide duration, the swimmers adapted the elbow and knee angles as well as the arm-leg coordination patterns during underwater recovery and propulsion, supporting the fact that expert swimmers exhibit reorganization of their whole behavior (i.e., limb angles and inter-limb coordination for the different phases of the cycle); (ii) our results also support the idea that seeking for flexibility towards behaviors that minimize *IVV* seems relevant for performance because economy was deteriorated in ‘maximal glide condition.

### 1. ‘Minimal glide’ coordination in breaststroke is effective and economical, and reveals functional adaptation

Although the swimmers were not able to glide less with the ‘minimal glide’ instruction, significant modifications of elbows and knees angles and arm-leg coordination were observed. As already reported by Seifert et al. [23], the target speed being quite slow, it is understandable that the swimmers were not able to reduce their glide duration. Indeed, Chollet et al. [15] showed that a ‘glide’ pattern of coordination was mostly observed for slow swimming speed, whereas ‘continuous’ and ‘superposition’ patterns of coordination are used at high swimming speed. However, in our study, the ‘minimal glide’ condition led the swimmers to significantly increase transitional velocity of the center of mass (i.e., lower deceleration between legs and arms propulsion), with a shape of velocity-time curve that resembles the ‘superposition’ pattern of coordination [15], [18], [19]. As the changes in limb angles and inter-limb coordination were not associated to significant difference in energy cost, these results suggest that minimizing the glide duration could be economical, according to the conclusion of Komar et al. [17] who stated that a coordination leading to continuous propulsion could be also effective. Indeed, minimizing glide avoids the drop of velocity between the leg and arm propulsions and thus no need for producing higher acceleration during arm propulsion in order to maintain a high mean velocity of the center of mass. It means that it is less energy consuming to maintain high mean velocity rather than to alternate high accelerations (e.g., during leg and arm propulsions) and high decelerations (e.g., during body glide). From there, the fact that swimmers were able to swim at the same target speed without extra energy cost of locomotion in both ‘freely-chosen’ and ‘minimal glide’ coordination represents an example of behavioral reorganization that is functional because for the same outcome, the swimmers exhibited adaptation of both limbs angle and inter-limb coordination, supporting neurobiological degeneracy property [40]–[43]. Degeneracy signifies that the swimmers were able to vary their behavior (structurally) without compromising performance outcome, providing evidence for the adaptive and functional role of movement and coordination pattern variability, in order to satisfy a set of constraints [4], [5], [44], [45]. In other words, the same function (assuming the same performance outcome) can be performed by different structures, each involving different joints and limbs, involving different coordination of those joints [41], [46]. In the present study, when swimmers were instructed to swim with ‘minimal glide’, they increased the relative duration of the leg propulsions without changing their performance, when compared to ‘freely-chosen’ coordination; i.e. the same instantaneous velocity was reached by the center of mass at the end of the leg propulsions for both ‘minimal glide’ and ‘freely-chosen’ coordination. Similarly, they started the arm propulsion earlier in the cycle without any significant change in the instantaneous velocity of the center of mass. Degeneracy was not only observed for the limbs movement, but also at the level of the inter-limb coordination. In particular, in ‘minimal glide’ condition, the swimmers showed the lowest standard deviation of elbow-knee pair's relative phase through the cycle, with lower values at the beginning of the cycle. It shows that the swimmers did not complete their underwater arm recovery (with the elbows fully extended) before starting leg propulsion.

### 2. ‘Maximal glide’ reveals behavioral reorganization

When swimmers were instructed to swim with ‘maximal glide’, they were able to satisfy the task-goal and thus exhibited a 45% increase in their glide duration. This modified glide was accompanied by higher energy cost and behavioral adaptations. According to Barbosa et al. [20], Nigg [47], Vilas-Boas et al. [48], higher energy cost has been commonly related to higher *IVV*. In the present study, extra energy expenditure was concomitant with higher deceleration of center of mass during the glide phase, which must be compensated by higher acceleration during the propulsive phase. In particular, the swimmers shortened their leg propulsion in a manner that was associated with higher acceleration of the center of mass, supporting that structural changes of the behavior were accompanied by functional reorganizations (i.e., change in the movement outcome, notably higher maximal velocity through legs propulsion). It shows that degeneracy is not only the ability of the structurally different components of a neurobiological system to perform the same task under certain conditions, but also the ability of these components to assume distinctly different roles in different conditions [40], [42]. It means that limbs movement and inter-limb coordination are not only regulated to keep as stable as possible the velocity of the center of mass, but that they are also organized to create high accelerations compensating high decelerations, in order to maintain the same speed both in ‘minimal’ and ‘maximal glide’ conditions. Finally, it is important to discuss the fact that the ‘maximal glide’ condition did not only lead to a reorganization of limb movement (e.g., leg propulsion) but influenced the whole behavioral adaptation. Notably, the higher deceleration due to longer glide duration was compensated by a more streamline body position that demands inter-limb coordination reorganization (e.g., flattest trunk inclination; higher arm extension during leg propulsion; better synchronization between the beginning of the legs propulsion and the end of the arms recovery). Thus, the ‘maximal glide’ condition cannot be summarized by a higher acceleration of the center of mass but induced also a minimization of the active drag [49]. The fact that some structures were less involved (e.g., trunk inclination) in ‘minimal’ than in ‘maximal glide’ condition, in order to minimize active drag, reflects another characteristic of neurobiological system degeneracy, which is called pluri-potentiality [41]. Indeed, pluri-potentiality corresponds to a surplus of structures for future situations, which means that during task performance, some limbs may be only slightly mobilized but may potentially be much more mobilized in the future [41]. Finally, the ‘maximal glide’ condition caused more than behavioral flexibility, in particular a reorganization of limbs movement and inter-limb coordination occurred and confirmed that swimmers exploit different characteristics of degeneracy to adapt their behavior in order to satisfy the task-goal.

## Conclusions

Instructing swimmers to use ‘maximal’ and ‘minimal glide’ conditions appeared as a fruitful way to assess their behavioral adaptability, because beyond than flexibility, these conditions caused whole behavioral reorganization in order to achieve the task-goal in a satisfying manner (i.e., without overly large increases in energy cost and/or *IVV*). Using a tether to pull or hold back, using a parachute to slow the swimmer can help him to learn how to have an inter-limb coordination more adaptable. We showed how swimmers were able to functionally adapt their limbs movement and inter-limb coordination during the different phases of the cycle in order to create an acceleration of the center of mass, keep the mean velocity of the center of mass as stable as possible. Finally, the assessment of behavioral adaptability is a promising way to approach dexterity and expertise, because it enables highlighting how the swimmers exhibit neurobiological degeneracy.
